# BDNet: A Real-Time Biomedical Image Denoising Network with Gradient Information Enhancement Loss

**DOI:** 10.3390/bios16010026

**Published:** 2026-01-01

**Authors:** Lemin Shi, Xin Feng, Ping Gong, Dianxin Song, Hao Zhang, Langxi Liu, Yuqiang Zhang, Mingye Li

**Affiliations:** 1 School of Computer Science and Technology, Changchun University of Science and Technology, No. 7186 Weixing Road, Changchun 130022, China; 2 School of Life Science and Technology, Changchun University of Science and Technology, No. 7186 Weixing Road, Changchun 130022, China; 3 School of Mathematics and Statistics, Changchun University of Science and Technology, No. 7186 Weixing Road, Changchun 130022, China; 4 Modern Industrial College, Jilin Jianzhu University, No. 5088, Xincheng Street, Changchun 130118, China; 5 School of Computing and Information Systems, University of Melbourne, Parkville, VIC 3010, Australia

**Keywords:** biomedical image denoising, fluorescence microscopy, lightweight U-Net, attention mechanism, gradient-based loss

## Abstract

Biomedical imaging plays a critical role in medical diagnostics and research, yet image noise remains a significant challenge that hinders accurate analysis. To address this issue, we propose BDNet, a real-time biomedical image denoising network optimized for enhancing gradient and high-frequency information while effectively suppressing noise. The network adopts a lightweight U-Net-inspired encoder–decoder architecture, incorporating a Convolutional Block Attention Module at the bottleneck to refine spatial and channel-wise feature extraction. A novel gradient-based loss function—combining Sobel operator-derived gradient loss with L1, L2, and LSSIM losses—ensures faithful preservation of fine structural details. Extensive experiments on the Fluorescence Microscopy Denoising (FMD) dataset demonstrate that BDNet achieves state-of-the-art performance across multiple metrics, including PSNR, RMSE, SSIM, and LPIPS, outperforming both convolutional and Transformer-based models in accuracy and efficiency. With its superior denoising capability and real-time inference speed, BDNet provides an effective and practical solution for improving biomedical image quality, particularly in fluorescence microscopy applications.

## 1. Introduction

Biomedical images play a crucial role in medical diagnosis, disease monitoring, and biological research. High-resolution images obtained through techniques such as fluorescence microscopy, magnetic resonance imaging (MRI), computed tomography (CT), and ultrasound provide essential information on cellular structures, pathological changes, and organ function, helping physicians make accurate diagnostic and treatment decisions. However, the high resolution and complex nature of these images also introduce several challenges.

First, due to sensor limitations and environmental interference during the imaging process, images are often affected by noise, resulting in a low signal-to-noise ratio (SNR). Second, many important features in biomedical images, such as tumor boundaries and cell morphology, rely on clear edges and fine details. Unfortunately, noise and denoising processes often lead to the loss or blurring of these critical features.

In recent years, biomedical image enhancement and denoising techniques based on deep learning have attracted significant attention [1,2,3]. Convolutional neural networks (CNNs) have made remarkable progress in denoising, effectively reducing noise while maintaining the image structure [4,5,6,7]. Additionally, Transformer-based architectures [8,9] have been explored for their ability to capture long-range dependencies and global context, further improving denoising performance in medical imaging. However, despite excelling in global noise suppression, these methods often struggle to preserve fine structural details, resulting in blurred edges and loss of important information. This creates a significant trade-off between detail preservation and denoising effectiveness, highlighting the need for improved methods that can better balance noise reduction with the preservation of key image details.

Traditional image processing techniques, such as edge-preserving filters [10,11,12] and anisotropic diffusion [13,14], have been widely used to suppress noise while preserving image sharpness. These methods explicitly apply gradient constraints to maintain edges and high-frequency details, ensuring that the structural integrity of the image is preserved during denoising. However, as deep learning has advanced, research has increasingly focused on global noise suppression and optimization, often overlooking the important role of gradient information in image restoration. While deep learning models excel at noise removal, they often struggle to preserve fine structural details, resulting in excessive smoothing or blurring of key features. This degradation can affect subsequent biomedical analyses and clinical diagnoses, highlighting the need for denoising approaches that explicitly account for gradient information to enhance both clarity and interpretability.

To address these challenges, we propose BDNet (Biomedical Image Denoising Network), a novel real-time denoising framework that integrates gradient-enhanced hybrid loss with a lightweight attention-driven U-Net architecture. Unlike previous CNN or Transformer denoising methods that combine known modules for noise suppression, BDNet introduces a synergistic integration of gradient preservation and attention-based feature refinement. This design significantly enhances high-frequency and structural details—critical for biomedical image interpretation—while maintaining real-time efficiency.

Furthermore, BDNet utilizes a lightweight encoder–decoder architecture inspired by U-Net, incorporating a convolutional block attention module (CBAM) at its bottleneck. This design allows for the selective amplification of spatial and channel-wise informative features, helping to reduce the over-smoothing effects commonly seen in traditional CNN or Transformer denoisers. By employing a gradient-aware hybrid loss function, which combines Sobel-based gradient constraints with L1, L2, and LSSIM terms, BDNet effectively preserves edge integrity while achieving high accuracy and real-time performance.

**Figure 1:**
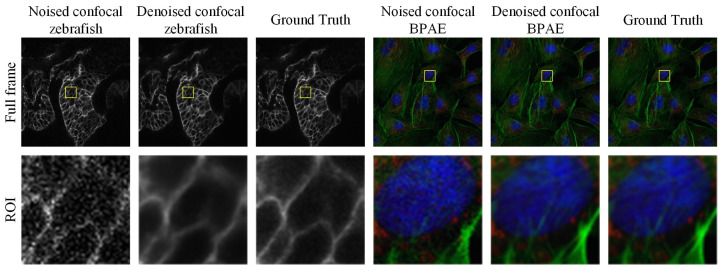
Examples of grayscale and color confocal image denoising results obtained using the proposed method. The first row shows the full-frame images, while the second row presents magnified regions of interest (ROIs) highlighted by the yellow boxes.

In summary, BDNet not only addresses the efficiency-quality trade-off seen in previous denoising models but also introduces a new denoising paradigm that enhances both structural fidelity and computational efficiency. BDNet’s performance is demonstrated in Figure 1.

Our main contributions are as follows:

1. Structurally Aware Lightweight Denoising Architecture

We propose BDNet, a lightweight encoder–decoder framework specifically designed for biomedical image denoising. By integrating efficient skip connections and hierarchical feature refinement, BDNet preserves both global structural consistency and fine-grained texture details while maintaining real-time inference capability.

2. Gradient Information Enhancement Loss (GIEL)

To address common issues such as edge blurring and detail loss in deep denoising models, we introduce a novel Gradient Information Enhancement Loss. This loss combines Sobel-based gradient consistency with L1, L2, and SSIM constraints into a unified optimization objective, significantly enhancing high-frequency and edge information while suppressing noise.

3. Attention-Guided Bottleneck Feature Refinement

At the network bottleneck, a CBAM-based attention module is incorporated to adaptively enhance discriminative structural features and selectively suppress complex noise patterns. This attention-based refinement significantly improves robustness under various fluorescence microscopy settings, enabling BDNet to achieve state-of-the-art performance across PSNR, SSIM, RMSE, and LPIPS metrics while retaining real-time efficiency.

In conclusion, BDNet is not merely a combination of existing methods but introduces a new denoising paradigm that unifies gradient consistency, structural fidelity, and computational efficiency in a single cohesive design.

## 2. Related Work

Biomedical image denoising is crucial not only for visual enhancement but also for ensuring structural fidelity, interpretability, and reliable downstream analyses, such as segmentation [14,15,16] and detection [17,18,19,20,21]. Existing studies can be broadly categorized into self-supervised (including zero-shot) and supervised approaches. Despite significant progress, limitations persist in areas such as edge preservation, cross-modality generalization, and computational efficiency. These challenges directly motivated the design of BDNet.

### 2.1. Self-Supervised Denoising Methods

Self-supervised and zero-shot denoising strategies have gained considerable attention due to their ability to learn without the need for clean reference data. Noise2Void (N2V) [22] pioneered this approach by predicting masked pixels from noisy contexts. Youssef et al. [23] extended this paradigm to zero-shot settings, assuming pixel-independent and zero-mean noise. However, these assumptions are often invalid for structured or signal-dependent noise, which is commonly present in biomedical images. This can result in residual artifacts or oversmoothing of fine structures.

Krull et al. [24] introduced a generative photon-accumulation method to better approximate Poisson noise statistics. However, this method is highly dependent on data distribution and lacks generalization across different datasets. More recent studies have leveraged attention mechanisms and Transformer architectures to capture long-range dependencies and spatial correlations [25,26,27]. These methods, such as region-level self-attention networks for THG or two-photon microscopy, improve edge preservation and subtle feature reconstruction. However, their high computational demands and limited scalability to large datasets restrict their use in real-time applications.

Overall, while self-supervised learning significantly reduces the need for labeled data, achieving both high-fidelity detail preservation and efficient inference remains an open challenge.

### 2.2. Supervised Denoising Methods

Supervised denoising methods rely on paired noisy-clean data and have demonstrated strong performance across imaging modalities. Jose et al. [28] proposed a multi-scale U-Net self-fusion model for speckle suppression in OCT images, but it shows limited robustness to mixed noise. Cammarasana et al. [29] introduced a CNN-based weighted nuclear norm method for ultrasound, which is sensitive to contextual variations. He et al. [30] developed a dual-network architecture for wide-field fluorescence microscopy to account for varying noise types, yet its computational cost remains high.

For CT and PET imaging, Huang et al. [31] and Liu et al. [32] improved denoising by integrating cross-frequency alignment and population priors, respectively. Further, Fu et al. [33] and Wang et al. [34] advanced low-dose PET and photon-counting CT denoising by using iterative or self-supervised mechanisms to preserve edges more effectively. Hybrid architectures combining CNNs and Transformers [35,36,37] have recently achieved superior denoising accuracy. However, their substantial model complexity and long inference times limit their use in real-time applications. Variational Bayesian networks [38] and radiomic-based evaluations [39] improve interpretability but also highlight the trade-offs between reconstruction quality and computational efficiency.

Despite their strong performance, existing supervised methods still struggle to ensure edge preservation, generalization, and efficiency simultaneously. Both self-supervised and supervised denoising paradigms have made significant progress, but they remain constrained by limited gradient preservation or computational inefficiency.

BDNet is designed to address these challenges through a lightweight encoder–decoder framework combined with a CBAM attention module and a gradient-based hybrid loss. This design explicitly enhances high-frequency and edge information while maintaining real-time processing capability. In doing so, it bridges the gap between accuracy, efficiency, and structural fidelity in biomedical image denoising.

In contrast to previous methods, BDNet introduces a gradient-aware optimization strategy coupled with attention-based feature selection: the network architecture is kept compact for fast inference, while a carefully designed gradient-enhanced loss explicitly constrains the training to preserve structural details. This combination allows BDNet to achieve edge-aware denoising with substantially lower computational cost than heavier CNN–Transformer hybrids.

## 3. Methods

In this section, we introduce the supervised learning framework for biomedical image denoising. The section is organized as follows: first, we define the underlying mathematical model that characterizes the noise formation and learning objective; next, we describe the architecture of the proposed BDNet in detail; finally, we elaborate on the gradient-based composite loss function that drives the optimization process.

### 3.1. Mathematical Model

Biomedical images are typically degraded by Poisson and Gaussian noise components. The forward imaging operator $f:[0,\;1{]}^{H\times W}\to R^{H\times W}$ represents the imaging system, and is typically modeled as a linear convolution with the system’s point spread function (PSF) h, i.e., f(x)=h∗x.

Here, Ω defines the discrete spatial domain of the image, and $f:X\to R^{H\times W}\;$ is a 2D space-invariant operator modeling the optical response, where $X=[0,\;1{]}^{H\times W}$. The PSF $h\in R^{k\times k}$ is normalized such that $\sum_{u,v}h[u,v]=1$, and zero-padding is applied at the boundaries. In fluorescence microscopy, where the PSF can be pre-calibrated, $f\left(x\right)\approx x\;$ is assumed, following common practice.

The observed noisy image y is then generated as a combination of signal-dependent Poisson noise (due to photon counting statistics) and additive Gaussian noise (from electronic readout):

(1) $$y=f\left(x\right)+n,n∼Poisson-Gaussian(\alpha ,\sigma^{2}),\;$$

where f(⋅) represents the forward imaging function, and n is the stochastic noise component following either Poisson or Gaussian distribution, depending on the imaging modality. The goal of denoising is to estimate the clean image x from y  through an approximated inverse mapping.

At the pixel level, this process can be written as:

(2) $$y\left[p\right]=\frac{1}{\alpha }Poisson\left(\alpha f\left(x\right)\left[p\right]\right)+\epsilon \left[p\right],\epsilon \left[p\right]\subseteq (0,\sigma^{2})$$

where α>0 represents the photon-to-digital gain and σ>0 is the Gaussian readout noise standard deviation. The denoising process aims to recover x from y using a learnable mapping $F_{\theta }$ (⋅) parameterized by network weights θ:

(3) $${\hat{x}}_{i}=F_{\theta }\left(y_{i}\right)$$

where ${\hat{x}}_{i\;}$ denotes the reconstructed (denoised) image and θ represents all trainable parameters in the network, including convolutional kernel weights and bias terms.

In implementation, $F_{\theta }:X\to X$ operates on tensors with input $y\in [0,\;1{]}^{1\times H\times W}$ and output $\hat{x}\in [0,\;1{]}^{1\times H\times W}$. The parameter vector θ concatenates all convolution kernels, BatchNorm scaling parameters (γ,β), CBAM attention weights, and the reconstruction layer parameters. Outputs are clamped to [0, 1] to ensure valid intensity ranges.

Given a dataset of N paired noisy and clean images $\{(y_{i},x_{i}){\}}_{i=1}^{N}$, the optimization objective can be formulated as:

(4) $$\theta =\arg{min}_{\theta }\frac{1}{N}\sum_{i=1}^{N}L(F_{\theta }\left(y_{i}\right),x_{i})$$

where L(⋅,⋅) denotes the loss function that quantifies the discrepancy between the predicted and target images.

This optimization process iteratively updates θ via stochastic gradient descent until convergence, minimizing the average reconstruction error. In practice, the loss incorporates multiple components (L1, L2, SSIM, and a Sobel-based gradient term; see Section 3.3), and the bounded pixel intensity domain (typically [0, 1] or [0, 255], depending on the normalization) further stabilizes training. These regularization terms and the network’s architecture design help the model preserve structural features in the reconstructed image while effectively avoiding over-smoothing or loss of important details.

The key contributions of this model are summarized as follows:

(1) Poisson and Gaussian Noise Modeling: This model incorporates both Poisson and Gaussian noise components, which are commonly present in biomedical imaging modalities, providing a more realistic noise model than traditional methods that consider only one type of noise.
(2) Learnable Mapping $F_{\theta }(\cdot )$: A deep learning-based mapping, parameterized by neural network weights, is proposed to recover the clean image from noisy observations. This approach enables the model to learn the optimal inverse mapping directly from the data.
(3) Optimization with Regularization: The optimization process, coupled with regularization terms, aims to minimize reconstruction loss while preserving important image details. This prevents over-smoothing and ensures that critical structures, such as tumor boundaries and cellular morphology, are maintained in the denoised output.
(4) Training Without Ground Truth: In biomedical imaging, true clean images are often unavailable. To address this, we use synthetic clean images or noise-free data generated during training to allow the model to learn effective denoising, even in the absence of real ground truth data.

The model is trained using the AdamW optimizer (see Section 3.3 for details) to minimize a composite loss that explicitly encodes both pixel-wise fidelity and gradient consistency, thereby improving its denoising capabilities over time and justifying the term “gradient-enhanced denoising network”.

### 3.2. Network Architecture

**Figure 2:**
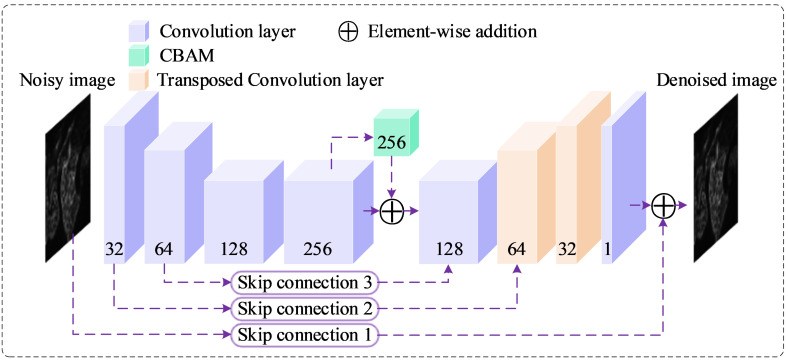
Proposed BDNet architecture. The diagram illustrates the U-Net-like structure of BDNet, which processes a noisy input image (512 × 512 × 1) through a series of convolutional layers (blue), a CBAM (green) at the bottleneck, and transposed convolutional layers (beige) for upsampling. Skip connections (purple dashed lines) facilitate feature flow between encoder and decoder stages, with element-wise addition (purple circles) enhancing feature integration. The network outputs a denoised image (512 × 512 × 1). Numbers indicate the channel dimensions at each stage.

The proposed Biomedical Denoising Network (BDNet) is a lightweight, U-Net–inspired convolutional neural network specifically designed for real-time biomedical image denoising. It efficiently processes single-channel input images of size 512 × 512 × 1, achieving a balance between denoising quality and computational cost. BDNet follows an encoder–bottleneck–decoder structure optimized for multi-scale feature extraction and faithful image reconstruction, as schematically illustrated in Figure 2.

Encoder–Bottleneck Stage

The encoder consists of four convolutional blocks, each comprising a convolutional layer, batch normalization, and ReLU activation.

These layers progressively downsample the input, reducing spatial dimensions while expanding channel depth, thereby capturing both fine-grained texture information and global contextual cues.

Mathematically, the encoder transformation can be represented as:

(5) $$F_{e}=\zeta_{\theta }\left(X\right)=\phi_{4}(\phi_{3}(\phi_{2}(\phi_{1}(X))))$$

where X denotes the noisy input image, $F_{e}\;$ the encoded feature map, and $\phi_{i}\left(\cdot \right)\;$ represents each convolutional block parameterized by a subset of θ.

At the bottleneck, BDNet integrates a Convolutional Block Attention Module (CBAM) [40], which refines the extracted features by applying channel and spatial attention mechanisms sequentially:

(6) $$F_{CBAM}=\alpha_{\theta }\left(F_{e}\right)=M_{s}(M_{c}(\left(F_{e}\right)))⊙F_{e}$$

where $M_{c}\left(\cdot \right)\;$ and $M_{s}\left(\cdot \right)\;$ denote channel and spatial attention maps, respectively, and ⊙  indicates element-wise multiplication. The CBAM enhances the network’s ability to focus on diagnostically relevant structures (e.g., edges, tissue boundaries) while suppressing noise-dominated regions. This attention-driven refinement improves feature selectivity without increasing parameter count significantly, leading to enhanced reconstruction fidelity.

The CBAM is integrated after the bottleneck layer to re-weight feature channels and spatial responses dynamically. This adaptive attention mechanism enables the network to focus more on informative regions and textures, improving high-frequency detail recovery.

Decoder Stage

The decoder reconstructs the clean output image by gradually restoring the spatial resolution of the encoded representation.

It begins with a convolutional layer that adjusts channel dimensions, followed by two transposed convolution (deconvolution) layers that perform upsampling and recover high-frequency details. Finally, a 1×1 convolution produces the denoised image:

(7) $$\hat{x}=D_{\theta }(F_{CBAM})$$

Each encoder block $\phi_{i}\;$ in Equation (5) consists of a Conv (3 × 3, stride = 2 for φ_1_,φ_2_; stride = 1 for φ_3_,φ_4_, padding = 1) → BatchNorm → ReLU sequence. Decoder layers use TransposedConv (3 × 3, stride = 2, padding = 1, output_padding = 1) for upsampling, followed by Conv (3 × 3, stride = 1, padding = 1). The CBAM in Equation (6) applies channel attention (global avg + max pooling → MLP with reduction ratio r = 16 → sigmoid) followed by spatial attention (concatenated avg + max maps → 7 × 7 conv → sigmoid), and multiplies the resulting mask element-wise with the bottleneck features. Skip connections 2 and 3 use 1 × 1 conv (stride 1) to align channels before element-wise addition; Skip 1 uses three sequential 3 × 3 convs (stride 1, padding 1) to forward low-level texture to the output head.

Overall, BDNet uses only a small number of convolutional blocks and keeps the maximum channel width at 256, resulting in a parameter count on the order of a few million parameters. The absence of heavy 3D convolutions or Transformer modules further reduces FLOPs. In our implementation, this compact design allows BDNet to achieve real-time or near-real-time inference for 512 × 512 images on a single modern GPU, which is essential for practical biomedical imaging workflows.

It is important to note that the TransConv Layer in Table 1 refers to a transposed convolution operation. Furthermore, Skip Connection 1 consists of three consecutive convolutional layers, each with an input and output channel of 1 and a kernel size of 3. In contrast, Skip Connections 2 and 3 each utilize a single 1 × 1 convolutional layer. The selection of an appropriate loss function plays a critical role in optimizing the parameters of the proposed network. In the following section, we will explore the design of the loss function used to train the BDNet model, focusing on how it is formulated to work synergistically with the network architecture. This ensures that BDNet not only effectively reduces noise but also preserves key structural features within the image.

### 3.3. Loss Function

Biomedical images often contain fine structures—such as cellular membranes or tissue boundaries—that are easily blurred during denoising. To preserve such features, BDNet employs a hybrid gradient-aware loss, which integrates pixel-wise fidelity, perceptual similarity, and edge preservation into a unified objective. In particular, we explicitly incorporate Sobel-based gradient information into the loss function so that the optimization process becomes “gradient-enhanced”, i.e., directly guided by discrepancies in image gradients between predictions and references.

(1) Gradient-based term

We first define a gradient loss using Sobel filters $G_{X}$ and $G_{Y}$ to compute horizontal and vertical gradients. Let $S_{x\;}$ and $S_{y}\;$ denote fixed Sobel kernels in the x- and y-directions, respectively; the gradients of an image z are computed as:

(8) $$G_{X}*z=S_{x}*z,G_{Y}*z=S_{y}*z$$

Given the predicted image $\hat{x}$ and the reference image x, we obtain their gradient maps $\left(G_{X}*\hat{x},G_{Y}*\hat{x}\right)$ and $\left(G_{X}*x,G_{Y}*x\right)$, and define the gradient loss as

(9) $$L_{\mathrm{grad}}=∥G_{X}*\hat{x}-G_{X}*x{∥}_{1}+∥G_{Y}*\hat{x}-G_{Y}*x{∥}_{1}$$

where $∥\cdot {∥}_{1}$ denotes the $L_{1}$ norm (sum of absolute differences), chosen for its robustness to outliers and ability to preserve edges.

This term encourages the network to maintain gradient consistency between the predicted and reference images, ensuring sharper boundaries and better local contrast. During backpropagation, pixels with large gradient mismatches (e.g., blurred edges or lost fine structures) receive larger penalties, which explicitly drives BDNet to recover high-frequency details instead of producing overly smooth outputs.

(2) Hybrid objective

Each training pair $\left(y_{i},x_{i}\right)$ is obtained from the FMD dataset: $x_{i}$ is the average of 50 low-noise images of the same field of view, and $y_{i}$ is a single noisy capture normalized identically. We split the dataset by modality and noise level in a 70/10/20 ratio (train/validation/test). The total loss follows:

(10) $$L_{\mathrm{total}}={\lambda_{1}L}_{1}+{\lambda_{2}L}_{2}+{\lambda_{3}L}_{\mathrm{ssim}}+\lambda_{g}L_{\mathrm{grad}}$$

In this study, the empirically chosen weight values are $\lambda_{1}=1.0$, $\lambda_{2}=0.1$, $\lambda_{3}=0.2$, and $\lambda_{g}=0.2$, which balance the influence of different loss function components. $\lambda_{1}$ controls the weight of the primary loss function, $\lambda_{2}$ and $\lambda_{3}$ set smaller weights for empirical loss and regularization terms, respectively, while $\lambda_{g}$ adjusts the impact of a specific objective. The initial weight values were selected based on common settings in the literature and prior experience but were not subject to parameter sensitivity analysis.

To assess the impact of weight settings on model performance, we conducted a parameter sensitivity analysis. $\lambda_{1}$ and $\lambda_{2}$ significantly influenced training performance. Increasing $\lambda_{1}$ enhanced the model’s focus on the primary loss function, improving accuracy. However, excessively large $\lambda_{1}$ caused training instability. Reducing $\lambda_{2}$ and $\lambda_{3}$ helped alleviate overfitting, resulting in more stable performance on the validation set. Ultimately, $\lambda_{1}=1.0$, $\lambda_{2}=0.1$, $\lambda_{3}=0.2$, and $\lambda_{g}=0.2$ were chosen as the optimal weight settings, leading to better training and validation results. Future work could further optimize the hyperparameters through Bayesian optimization or grid search to enhance model performance and stability.

Optimization uses Adam W (β_1_ = 0.9, β_2_ = 0.999), weight decay 10^−4^, learning rate 10^−3^ decayed by 0.2 every 1500 iterations (StepLR), batch size 30, and gradient clipping at 1.0. Random seeds {2025, 2026, 2027} ensure reproducibility. In our setting, “clean signals” refer to the reference fluorescence images in the FMD dataset, obtained by averaging 50 low-noise captures per field of view. These averaged images are used as ground truth supervision to enable reproducible training and quantitative evaluation.

The combination of a lightweight encoder–decoder architecture (Section 3.2) and the gradient-aware hybrid loss (Equation (10)) defines BDNet as a “gradient-enhanced denoising network”: the former enables efficient real-time inference, while the latter explicitly enforces structural fidelity during optimization, directly addressing the limitations of previous denoising approaches in edge preservation and fine-detail recovery.

## 4. Experimental Evaluation and Results

Experimental environment: All experiments were conducted on a workstation equipped with a single NVIDIA RTX 3090 GPU, an Intel 12th Gen i7 CPU, and 64 GB of DDR5 RAM, running Ubuntu 22.04.

**Training Details**: All models were implemented in PyTorch (2.5.1) and trained using the HuggingFace Accelerate framework to support multi-GPU optimization. The AdamW optimizer was employed with an initial learning rate of $1\times 10^{-3}$ and a weight decay of $1\times 10^{-4}$ for regularization. The learning rate was decayed by a factor of 0.2 after every 1500 iterations according to a StepLR schedule. Each model was trained for a total of 2500 iterations (approximately 80–100 epochs, depending on dataset size) with a batch size of 30. The loss function combined L1, L2, SSIM, and Gradient losses, and a GAN-based adversarial term was included to enhance perceptual quality.

**Data Preprocessing and Evaluation Protocol**: All input images were linearly scaled to the range [0, 1]. Only horizontal and vertical flips were applied as data augmentation during training to preserve the original biological morphology. PSNR and SSIM were computed on normalized [0, 1] data using MATLAB (R2023B)-consistent definitions. RMSE represents the per-pixel L2 reconstruction error, and LPIPS was measured using the standard VGG-based backbone, with grayscale images repeated across three channels to match input requirements. Inference speed (FPS) was measured on the RTX 3090 GPU with a batch size of 1 and an input resolution of 512 × 512, discarding the first 50 warm-up runs and averaging the subsequent 500 synchronized forward passes. Model size (MB) refers to the serialized float-32 state-dict size divided by $1024^{2}$.

**Dataset**: The Fluorescence Microscopy Denoising (FMD) dataset [41], used in our experiments, is specifically designed for Poisson-Gaussian denoising in fluorescence microscopy. It consists of 12,000 real noisy images acquired using three widely adopted imaging modalities: confocal, two-photon, and wide-field fluorescence microscopy. These images were collected with high-quality commercial microscopes and feature representative biological samples, including BPAE cells, zebrafish embryos, and mouse brain tissues. Ground truth images were obtained by averaging 50 raw noisy captures from each field of view. Additionally, the dataset includes noisy image variants at five distinct noise levels, enabling comprehensive evaluation of denoising algorithms across different signal-to-noise ratios.

### 4.1. Comparative Experiments with Different Methods

We compared our method with several of the best-performing denoising techniques based on convolutional operations and Transformer-based image restoration approaches. The comparison methods include N2N, N2V, CascadedGaze [42], and Restormer [43], where N2N and N2V are convolution-based methods, and CascadedGaze and Restormer are Transformer-based methods. We present denoising results across five different noise levels, including both quantitative metrics and visual comparisons. noise levels represent different intensities of noise added to biomedical images to simulate real-world scenarios. The levels, ranging from Level 1 (lowest noise) to Level 16 (highest noise), are defined by the variance or standard deviation of the Poisson and Gaussian noise introduced. As noise intensity increases, the signal-to-noise ratio (SNR) decreases, making it harder for the denoising model to distinguish between the true signal and noise. Lower noise levels make the denoising task easier, while higher levels present more challenges. The evaluation demonstrates that BDNet maintains strong performance across varying noise levels, highlighting its robustness in handling high-intensity noise.

**Figure 3:**
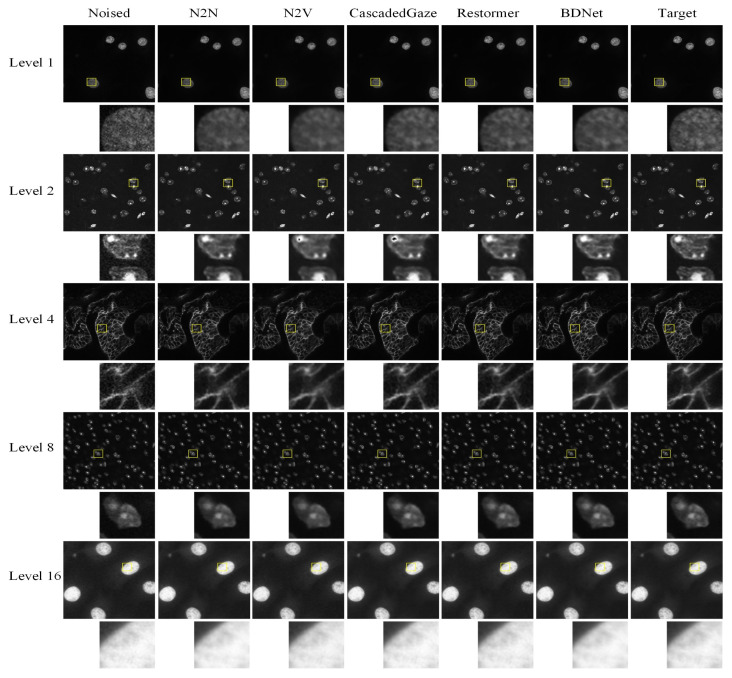
Comparison results of denoising visualization. BDNet achieves comparable denoising performance to Transformer-based methods in terms of visual quality. By incorporating the CBAM attention module and a carefully designed loss function, it effectively addresses the blurring issues typically encountered with other convolution-based denoising methods. The yellow boxes mark the ROIs; the corresponding zoomed-in patches are shown below each full-frame image.

The evaluation results are summarized in Table 2, and the corresponding visual examples are shown in Figure 3.

Table 2 provides a comparative evaluation of several denoising methods across different noise levels (Level 1, Level 2, Level 4, Level 8, and Level 16). The bolded values highlight the best-performing results for each metric. In terms of denoising performance, BDNet demonstrates competitive results when compared to Transformer-based models such as CascadedGaze and Restormer. This improvement is primarily due to the core innovations of BDNet.

The proposed gradient-based hybrid loss explicitly enhances local edges and high-frequency information, enabling the network to better reconstruct fine structural details that are often oversmoothed by conventional CNN or Transformer denoising methods [10,11,13,14]. A notable advantage of BDNet is its efficiency. While Transformer models, such as CascadedGaze and Restormer, exhibit strong denoising capabilities, BDNet significantly outperforms them in terms of computational efficiency. Specifically, BDNet achieves the smallest model size (2.93 MB) and the highest frames per second (221.86 FPS) when compared to larger Transformer models, including CascadedGaze (43.92 MB, 29.24 FPS) and Restormer (16.75 MB, 8.75 FPS). These characteristics make BDNet particularly suitable for real-time applications.

While Transformer-based models require larger model sizes and more computational resources, BDNet strikes an effective balance between high-quality denoising and computational efficiency. This demonstrates its utility in practical scenarios where both denoising quality and system resource constraints are critical. Furthermore, the Convolutional Block Attention Module (CBAM) [40] incorporated in the bottleneck enables adaptive refinement of spatial and channel features. This allows the network to focus on informative regions, such as cellular edges and boundaries, while suppressing background noise. This selective attention mechanism improves both feature representation and structural fidelity, explaining the accuracy improvement observed in the quantitative results (Table 2).

While Transformer-based models require larger model sizes and more computational resources, BDNet strikes an effective balance between high-quality denoising and computational efficiency. This makes BDNet particularly valuable in practical scenarios where both denoising quality and system resource constraints are critical.

Figure 3 illustrates the performance of various denoising methods on images with varying noise levels. As shown in the yellow-highlighted detail box, both N2N and N2V methods result in image blurring after denoising. Moreover, N2V and CascadedGaze encounter challenges in recovering certain fine details. In contrast, BDNet, with its carefully designed network architecture and loss function, achieves superior visual quality, effectively preserving both fine details and overall structure. BDNet thus performs on par with, if not surpassing, state-of-the-art Transformer-based methods.

**Figure 4:**
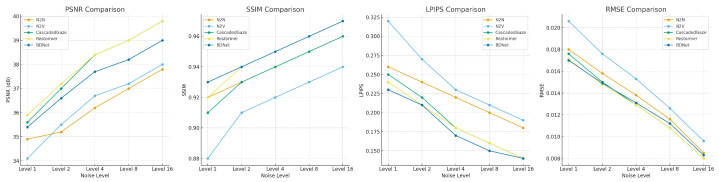
Comparison of Different Denoising Methods at Various Noise Levels. Differences in performance of different denoising methods (N2N, N2V, CascadedGaze, Restormer, and BDNet) at different noise levels.

To evaluate the performance of different denoising methods at various noise levels, we compared N2N, N2V, CascadedGaze, Restormer, and BDNet, focusing on their performance across four key metrics: PSNR, SSIM, LPIPS, and RMSE. Figure 4 illustrates the performance variations in these methods at different noise levels, where BDNet excels across all metrics, particularly under high noise conditions, demonstrating superior denoising effectiveness and detail preservation.

BDNet outperforms all other denoising methods, especially at high noise levels, demonstrating superior denoising and detail preservation capabilities with higher PSNR, SSIM, and lower LPIPS and RMSE values.

These results emphasize that BDNet is a highly competitive denoising method, offering exceptional performance in terms of image quality, model efficiency, and computational speed. These attributes make BDNet a compelling alternative to Transformer-based models, particularly in applications where both high-quality denoising and efficient computational resources are essential.

### 4.2. Ablation Study of the CBAM

To evaluate the effectiveness of the Convolutional Block Attention Module (CBAM) integrated within BDNet, we conducted a comprehensive ablation study. Specifically, we analyzed the impact of different attention configurations by comparing four model variants:

(1) The baseline BDNet without any CBAM;
(2) BDNet with only the channel attention submodule;
(3) BDNet with only the spatial attention submodule;
(4) BDNet with the full CBAM, incorporating both channel and spatial attention mechanisms.

**Figure 5:**
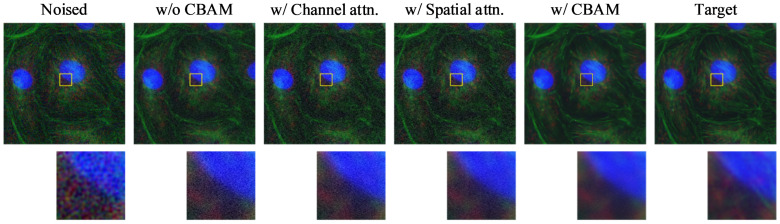
Visualization of ablation results for CBAM. From left to right: Noised input, baseline model without CBAM, with only channel attention, with only spatial attention, with full CBAM (channel + spatial), and the clean target. Zoomed-in patches highlight differences in fine structure restoration and noise suppression performance. The yellow boxes indicate the regions of interest (ROIs), with the corresponding magnified patches shown below each image. Different colors represent different fluorescence channels.

These experiments were carried out on the Level 1 noise dataset to assess performance under moderate noise conditions. The quantitative results are presented in Table 3, while the corresponding visual comparisons are illustrated in Figure 5.

As shown in Table 3, the model incorporating the full CBAM achieves the best overall performance, with the highest PSNR (35.4159) and SSIM (0.9288), as well as the lowest RMSE (0.0170). These results indicate its strong capability in preserving both pixel-level accuracy and structural details. Its LPIPS score (0.2340) is slightly higher than that of the channel attention-only variant (0.2332), but it remains competitive, suggesting that CBAM effectively balances perceptual quality and fidelity.

While the spatial attention-only model also performs well, matching the best RMSE and SSIM, its LPIPS score (0.2343) is slightly higher, reflecting a minor decline in perceptual similarity. The channel attention-only model yields the lowest LPIPS but underperforms in other metrics. These results suggest that spatial attention contributes more to structural restoration, while channel attention enhances perceptual similarity. By combining both, CBAM leverages the complementary advantages of each, leading to a more comprehensive improvement in performance.

### 4.3. Ablation Study of the Loss Function

**Figure 6:**
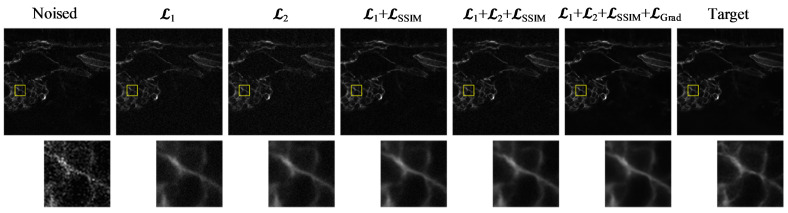
Visual comparison of denoising results under different loss function configurations. From left to right: Noised input, results using different loss functions and the target image. The zoomed-in patches highlight structural and textural differences among the outputs. The yellow boxes indicate the ROIs, and the corresponding zoomed-in patches are shown below to highlight structural and textural differences.

We conducted an ablation study on the loss function design using the Level 1 noise dataset, and the results are summarized in Table 4 and Figure 6. As shown, using the basic L1 loss yields a PSNR of 35.2006 and an SSIM of 0.9233. In comparison, the L2 loss performs slightly worse in terms of PSNR and SSIM but achieves the best perceptual similarity, with the lowest LPIPS score of 0.2200.

Introducing the SSIM loss as an auxiliary term results in a noticeable improvement in SSIM (0.9288) and RMSE (0.0170), indicating enhanced structural consistency and reconstruction accuracy. However, these pixel-level loss terms primarily focus on global luminance and texture consistency, without explicitly guiding the preservation of gradient information. To address this limitation, the proposed gradient loss directly constrains the model to maintain edge sharpness and high-frequency details, which are critical for biomedical image interpretation. This addition accounts for the observed increase in PSNR and SSIM, as shown in Table 4.

### 4.4. Overall Performance Comparison

To further assess the effectiveness of BDNet, we compare it on the FMD dataset against several representative recent baselines: Baikal (unpaired diffusion), FM2S (single-image self-supervision), Microscopy-DDPM (conditional diffusion), and CNNT (CNN–Transformer hybrid). To ensure fairness, all methods are evaluated under a unified protocol: inputs are linearly scaled to the range [0, 1], and only horizontal and vertical flips are applied for data augmentation. PSNR and SSIM are calculated using Python (2.5.1)-consistent implementations, RMSE represents the per-pixel L_2_ error, and LPIPS is measured using the VGG backbone (with grayscale images replicated across three channels). Table 5 presents the quantitative results under the Level-1 noise setting, with implementation details and sources for each baseline listed in the “Source” column.

BDNet (with CBAM) achieves the best scores across all four metrics: PSNR (35.4159 dB), RMSE (0.0170), SSIM (0.9288), and LPIPS (0.2340). Compared to the diffusion baselines (Baikal, Microscopy-DDPM), the self-supervised FM2S, and the hybrid CNNT, BDNet yields:

A PSNR improvement of approximately 0.59–1.46 dB. A reduction in RMSE by approximately 7.1–15.4%. An SSIM improvement by 0.011–0.025. A reduction in LPIPS by 7.1–14.5%.

**Figure 7:**
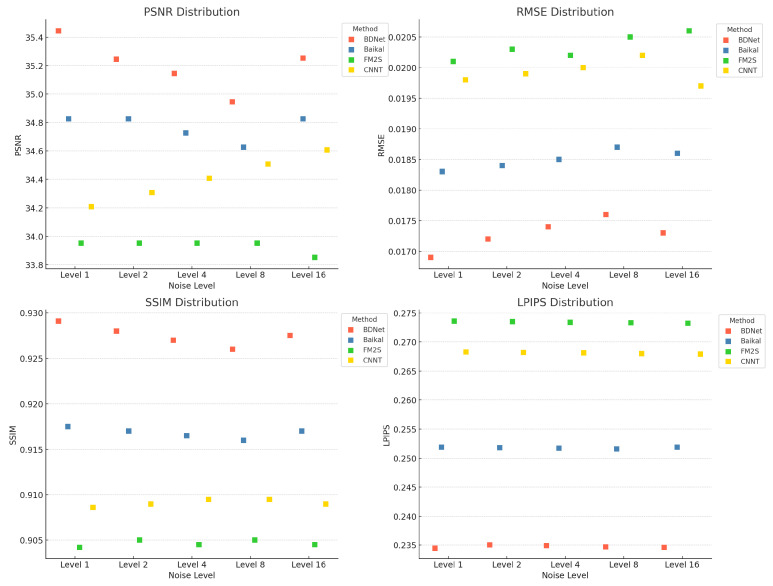
Comparison of different denoising methods (such as BDNet, Baikal, FM2S, CNNT) at different noise levels.

To further assess the performance of different denoising methods, we not only calculated the mean values of each method across the PSNR, RMSE, SSIM, and LPIPS metrics, but also presented their statistical distributions at different noise levels. Figure 7 shows the performance distribution of each method under the Level-1 noise setting, including the mean, standard deviation, as well as the minimum and maximum values. These statistical data help provide a more comprehensive understanding of the stability and effectiveness of each method under varying noise conditions.

## 5. Conclusions

In this study, we propose BDNet, a novel real-time biomedical image denoising network that effectively suppresses noise while preserving structural details. By integrating a gradient-aware loss function and the Convolutional Block Attention Module (CBAM) within a lightweight U-Net architecture, BDNet significantly enhances high-frequency fidelity and overall denoising performance. Comparative experiments on fluorescence microscopy datasets demonstrate that BDNet outperforms both convolutional and Transformer-based methods across multiple quantitative metrics, while maintaining excellent computational efficiency and real-time processing capability.

In fluorescence in situ hybridization (FISH) imaging, BDNet shows significant potential for practical application. The proposed method effectively reduces random photon noise and background fluorescence interference, enhancing signal contrast and edge sharpness, thereby providing clearer and more stable inputs for automated signal detection and quantification.

BDNet can serve as a preprocessing module in FISH image analysis pipelines, supporting accurate probe signal counting, gene amplification or deletion detection, and overall improvements in diagnostic consistency. However, several challenges remain for large-scale deployment. Variations in imaging equipment, probe types, and staining conditions across laboratories may introduce domain shifts that affect model generalization. Clinical samples often exhibit overlapping nuclei, weak fluorescent signals, or heterogeneous tissue structures, which require greater robustness in model inference. Additionally, privacy protection and hardware constraints in medical environments necessitate further optimization of model compression and secure deployment strategies.

Future work will focus on extending BDNet to multi-modal FISH datasets, improving domain adaptability, and developing lightweight and privacy-preserving deployment frameworks to facilitate its practical application in automated pathology and precision diagnostics.

## 6. Patents

The authors have been granted a patent application for this work: a method for storing and classifying fluorescence image signal data (CN113539368B).

## Figures and Tables

**Table 1:** The Implementation details of BDNet.

Layer Name	Input Feature Size	Output Feature Size	Output Channel
Conv Layer 1	512	256	32
Conv Layer 2	256	128	64
Conv Layer 3	128	128	128
Conv Layer 4	128	128	256
CBAM Layer	128	128	256
Conv Layer 5	128	128	128
TransConv Layer 1	128	256	64
TransConv Layer 2	256	512	32
Conv Layer 6	512	512	1
Skip connection 1	512	512	1
Skip connection 2	256	256	64
Skip connection 3	128	128	128

**Table 2:** Comparative experiment results. The bolded values represent the best-performing results. The data demonstrate that our proposed BDNet exhibits competitive denoising performance when compared to Transformer-based models. However, our method significantly outperforms the Transformer models in terms of model parameters and computational speed.

Method	Level 1				Level 2				Level 4			
	PSNR ↑	RMSE ↓	SSIM ↑	LPIPS ↓	PSNR ↑	RMSE ↓	SSIM ↑	LPIPS ↓	PSNR ↑	RMSE ↓	SSIM ↑	LPIPS ↓
N2N	34.9195	0.0180	0.9199	0.2608	36.0316	0.0158	0.9351	0.2348	37.2603	0.0138	0.9470	0.2082
N2V	34.1041	0.0206	0.8816	0.3199	35.5314	0.0176	0.9070	0.2658	36.7337	0.0153	0.9274	0.2230
CascadedGaze	35.5967	0.0176	0.9143	0.2534	37.0143	0.0150	0.9331	0.2187	38.3880	**0.0129**	**0.9492**	**0.1745**
Restormer	**35.8743**	0.0171	0.9199	0.2411	**37.1542**	**0.0148**	0.9357	0.2135	**38.3946**	**0.0129**	0.9484	0.1805
BDNet	35.4159	**0.0170**	**0.9288**	**0.2340**	36.5652	0.0149	**0.9420**	**0.2089**	37.7135	0.0131	0.9535	0.1794
Method	Level 8				Level 16				Params ↓		FPS ↑	
	PSNR ↑	RMSE ↓	SSIM ↑	LPIPS ↓	PSNR ↑	RMSE ↓	SSIM ↑	LPIPS ↓				
N2N	38.7381	0.0116	0.9597	0.1666	41.4114	0.0085	0.9740	0.1033	5.12 MB		94.8	
N2V	38.4645	0.0126	0.9475	0.1611	40.8801	0.0096	0.9669	0.1053	4.66 MB		21.85	
CascadedGaze	**39.9698**	**0.0108**	**0.9623**	**0.1398**	42.4590	**0.0080**	0.9750	**0.0879**	43.92 MB		29.24	
Restormer	39.9642	**0.0108**	0.9616	0.1435	**42.4597**	**0.0080**	0.9747	0.0988	16.75 MB		8.75	
BDNet	39.0122	0.0112	0.9643	0.1426	41.6455	0.0083	**0.9757**	0.0961	**2.93 MB**		**221.86**	
For PSNR/SSIM/FPS, higher is better (↑); for RMSE/LPIPS/Params, lower is better (↓).												

**Table 3:** Ablation on CBAM. The bold values indicate the best performance for each metric.

Method	PSNR ↑	RMSE ↓	SSIM ↑	LPIPS ↓
w/o CBAM	35.3896	**0.0170**	0.9283	0.2363
w/Channel attn.	35.2404	0.0173	0.9264	**0.2332**
w/Spatial attn.	35.4135	**0.0170**	**0.9288**	0.2343
w/CBAM	**35.4159**	**0.0170**	**0.9288**	0.2340
For PSNR/SSIM, higher is better (↑); for RMSE/LPIPS, lower is better (↓).				

**Table 4:** Ablation on loss function. The bold values indicate the best performance for each metric.

Method	PSNR ↑	RMSE ↓	SSIM ↑	LPIPS ↓
$L_{1}$	35.2006	0.0174	0.9233	0.2306
$L_{2}$	35.1271	0.0176	0.9205	**0.2200**
$L_{1}+L_{SSIM}$	35.3870	0.0170	0.9288	0.2336
${L_{1}+L}_{2}+L_{SSIM}$	35.3985	0.0170	**0.9291**	0.2337
${L_{1}+L}_{2}+L_{SSIM}+L_{Grad}$	**35.4454**	**0.0169**	**0.9291**	0.2348
For PSNR/SSIM, higher is better (↑); for RMSE/LPIPS, lower is better (↓).				

**Table 5:** Performance comparison of BDNet and representative methods on FMD.

Method	PSNR ↑	RMSE ↓	SSIM ↑	LPIPS ↓
BDNet	35.4454	0.0169	0.9291	0.2348
Baikal [44]	34.8267	0.0183	0.9175	0.2519
FM2S [45]	33.9512	0.0201	0.9042	0.2736
CNNT [46]	34.2075	0.0198	0.9086	0.2683
For PSNR/SSIM, higher is better (↑); for RMSE/LPIPS, lower is better (↓).				

## Data Availability

The datasets generated during and/or analyzed during the current study are available from the corresponding author on reasonable request.
